# Risk of bias in observational studies using routinely collected data of comparative effectiveness research: a meta-research study

**DOI:** 10.1186/s12916-021-02151-w

**Published:** 2021-11-23

**Authors:** Van Thu Nguyen, Mishelle Engleton, Mauricia Davison, Philippe Ravaud, Raphael Porcher, Isabelle Boutron

**Affiliations:** 1 Centre of Research Epidemiology and Statistics (CRESS), Inserm, Université de Paris, F-75004 Paris, France; 2 Meta-Research Innovation Centre at Stanford (METRICS), Stanford University, 1265 Welch Rd, Stanford, CA 94305 USA; 3 Cochrane France, AP-HP, 75004 Paris, France; 4 Centre d’Epidémiologie Clinique, Hôpital Hôtel Dieu, Assistance Publique des Hôpitaux de Paris (APHP), F-75004 Paris, France

**Keywords:** Observational studies, Routinely collected data, Emulated trial, Meta-research, Risk of bias

## Abstract

**Background:**

To assess the completeness of reporting, research transparency practices, and risk of selection and immortal bias in observational studies using routinely collected data for comparative effectiveness research.

**Method:**

We performed a meta-research study by searching PubMed for comparative effectiveness observational studies evaluating therapeutic interventions using routinely collected data published in high impact factor journals from 01/06/2018 to 30/06/2020. We assessed the reporting of the study design (i.e., eligibility, treatment assignment, and the start of follow-up). The risk of selection bias and immortal time bias was determined by assessing if the time of eligibility, the treatment assignment, and the start of follow-up were synchronized to mimic the randomization following the target trial emulation framework.

**Result:**

Seventy-seven articles were identified. Most studies evaluated pharmacological treatments (69%) with a median sample size of 24,000 individuals. In total, 20% of articles inadequately reported essential information of the study design. One-third of the articles (*n* = 25, 33%) raised some concerns because of unclear reporting (*n* = 6, 8%) or were at high risk of selection bias and/or immortal time bias (*n* = 19, 25%). Only five articles (25%) described a solution to mitigate these biases. Six articles (31%) discussed these biases in the limitations section.

**Conclusion:**

Reporting of essential information of study design in observational studies remained suboptimal. Selection bias and immortal time bias were common methodological issues that researchers and physicians should be aware of when interpreting the results of observational studies using routinely collected data.

**Supplementary Information:**

The online version contains supplementary material available at 10.1186/s12916-021-02151-w.

## Background

Though randomized control trials (RCTs) are considered to provide the best evidence in comparative effectiveness research (CER), they also have some limitations [1, 2]. They can often be resource-intensive and time-consuming. As such, RCTs may not be able to detect effects on long-term outcomes or rare events [3–5]. Observational studies using routinely collected data have been used to complement RCTs [5–8]. Routinely collected health data (RCD) are generated from the daily operations of healthcare systems, recorded without a priori research question [6]. A broad range of sources (e.g., disease registries, health administrative data, quality/safety surveillance databases, electronic health records, and pharmacy data) hosts such routinely collected data and contains both drug exposure and clinical outcomes to be used to provide evidence on treatment effectiveness.

However, observational studies are limited by their susceptibility to bias [5, 9–11]. Hernán et al. published a framework for using observational data to emulate a target trial, a hypothetical pragmatic trial [4, 12]. The framework suggested researcher explicitly specifying key components of this hypothetical trial such as eligibility criteria, treatment assignment, and the start of follow-up. The time when patients fulfill the eligibility criteria is assigned to one of the treatment strategies (i.e., fulfill the criteria to be classified as exposure or control), and starting the follow-up should be aligned to mimic the randomization process in an RCT [3, 4, 12]. By avoiding methodological pitfalls, this approach reduces the risk of bias of the effect estimate and hence produces more reliable results [13]. Cochrane has adopted this framework in the assessment of the risk of bias for non-randomized intervention studies [14].

This study aimed to assess the completeness of reporting essential information of study design and risk of bias due to failure to mimic the randomization in observational studies using routinely collected data for comparative effectiveness research. We did not aim to assess the extent that the bias could influence the conclusion of the included studies. After systematically reviewing the reporting and conducting of observational studies, we propose a checklist to help readers and reviewers to identify common methodological pitfalls of observational studies.

## Methods

### Study design

We conducted a meta-research study and reviewed the comparative effectiveness observational studies evaluating the therapeutic interventions with the use of routinely collected data published in high impact factor journals. We followed the Preferred Reporting Items for Systematic Reviews and Meta-Analyses (PRISMA) guidelines [15].

### Search strategy

We identified a convenience sample of the 7 highest impact factor journals of the InCites Journal Citation Reports categories medicine, general, and internal (*New England Journal of Medicine*, *Lancet*, *JAMA*, *BMJ*, *Annals of Internal Medicine*, *BMC Medicine*, and *PLoS Medicine*) and 3 highest impact factor journals in endocrinology and metabolism (*Lancet Diabetes & Endocrinology*, *Diabetes Care*, and *Diabetes*) and cardiac and cardiovascular systems (*European Heart Journal*, *Journal of American College of Cardiology*, and *Circulation*) that cover research on high prevalent diseases.

As all these ten journals were indexed on PubMed, we conducted a search on PubMed to identify the observational studies evaluating a comparative effectiveness question. To reflect contemporary reporting practices and methodological conduct, the search was narrowed to studies published between 01/06/2018 and 30/06/2020. The full search strategy is presented in Additional file 1: Table S1.

### Eligibility criteria

We included cohort studies which evaluated a therapeutic intervention by using RCD [6]. Studies were eligible for inclusion if they (1) evaluated a therapeutic intervention, defined as a treatment-related to healing a disease, i.e., pharmaceuticals, surgery; (2) used RCD as the data source; and (3) answered a comparative effectiveness question, i.e., research aiming to identify which interventions work best for improving health. Studies that did not answer CER questions, studies without an abstract, and retracted papers were excluded. The inclusion and exclusion criteria for study selection are provided in Additional file 1: Table S2.

### Study screening and selection

One reviewer (ME) screened all the titles and abstracts of the studies retrieved. A second reviewer (VNT) screened a sample of 775 (57%) of 1357 articles excluded by ME. There was good agreement between the two reviewers with only 1 conflict. Then, each of the full texts was assessed by two of three reviewers (ME, VNT, MD) to ensure the eligibility of the study for data extraction. All conflicts were resolved through discussion, and a third reviewer was available to adjudicate. Literature search results were imported into Mendeley (https://www.mendeley.com) to store, organize, and manage all references. The screening process was aided by the use of the Rayyan software [16].

### Data extraction

Data from each article were extracted independently by two of the three reviewers (ME, VTN, and MD) using a standardized form created based on the framework for emulating a target trial proposed by Hernán et al. and RECORD-PE reporting guideline for observational studies using routinely collected data for pharmacoepidemiology [4, 12, 14, 17]. The form was initially piloted and refined throughout the process (Additional file 1: Table S3 – data extraction form and Additional file 1: Table S4 – explanation of data items). Any disagreement was discussed with senior researchers (RP, IB) to reach a consensus. The following data were extracted from the selected papers:

1. Study characteristics: title, year of publication, author, location of the corresponding author, name of the journal, study design (longitudinal study), treatment type, comparator, funding source (i.e., public, private funding), and data source
2. Research transparency practices: use of reporting guidelines, access to codes and algorithms to classify exposures and outcomes, and data sharing policy
3. Reporting of essential items:
  - Diagram to illustrate the study design (i.e., describing the time of eligibility, treatment assignment, and follow-up).
  - Eligibility criteria, and particularly whether individuals with contraindication to one of the evaluated treatments, were explicitly excluded as in an RCT.
  - Methods used to adjust for confounding (i.e., regression, propensity score, inverse probability weighting).
  - Causal contrast of interest (i.e., intention-to-treat effect, per-protocol effect).
  - Time points of eligibility (i.e., when individuals were evaluated regarding their eligibility), treatment assignment (i.e., when individuals were classified to one of the treatment groups), and the start of follow-up (i.e., when individuals started outcome assessment).
4. After determining the time points of eligibility, treatment assignment, and the start of follow-up, we assessed if these time points were aligned to avoid bias. We identified the type of bias that might arise when they were not aligned (Table 1) and whether the authors described a solution to address bias.

**Table 1:** Situations when time points of eligibility, treatment assignment, and the start of follow-up are not aligned

Situations	Type of bias that might arise
*a. Follow-up starts after eligibility criteria completion and treatment assignment.* This situation happens when the follow-up starts after eligible individuals have started the treatment. The follow-up time is left-truncated, and individuals who experience early outcomes after starting treatment are not captured.	Prevalent user bias
*b. Follow-up starts at eligibility but after treatment assignment.* This situation happens when the follow-up starts after individuals have started the treatments, which means that the follow-up time is left-truncated. Additionally, individuals are selected based on post-treatment criteria (e.g., individuals have no outcome that occurred before the start of the follow-up).	Prevalent user bias and selection bias due to post-treatment eligibility
*c. Follow-up starts before treatment assignment and eligibility.* This situation happens when individuals need to meet the eligibility criteria after the follow-up has started and individuals have started treatments. For example, patients have to receive at least 2 consecutive prescriptions of treatment to be included in the analysis, but follow-up starts from the first prescription. Those who have an outcome within this time are excluded from the analysis leading to immortal time bias, or those who stop treatment after the first prescription are excluded leading to selection bias.	Immortal time bias and selection bias due to post-treatment eligibility
*d. Follow-up starts at eligibility, but treatment is assigned later.* This situation happens in two cases: 1) When there is a grace period, a period from when individuals meet the eligibility to when they start treatments. For example, a study compares no antibiotic use with initiation of antibiotic use within 7 days since diagnosis of urinary tract infection. If an individual starts antibiotics on day 7, it means that they have survived for 7 days leading to immortal time bias. 2) When individuals have to use the treatment for a given period to be classified in the exposed group. For example, individuals have to fill three consecutive prescriptions of aspirin to be classified as an aspirin user group, and non-aspirin users, otherwise. This also leads to immortal time bias. Another issue that might arise from this situation is the risk of misclassification of treatment. For instance, in the example of initiating antibiotics within 7 days since diagnosis, if the individual has an outcome before day 7 and has not started the antibiotic, we are uncertain to classify her/him to the no-antibiotic user or antibiotic user.	Immortal time bias and misclassification of treatment

### Data synthesis

Categorical data were summarized using frequencies and percentages. Interrater reliability was tested using Cohen’s kappa [18]. Descriptive analysis was completed in R (version 4.0.2).

### Data sharing

Data of this study will be available on Zenodo after the publication of the article.

### Patient involvement

Patients and public members were not involved in this study.

## Results

### Study characteristics

**Fig. 1:**
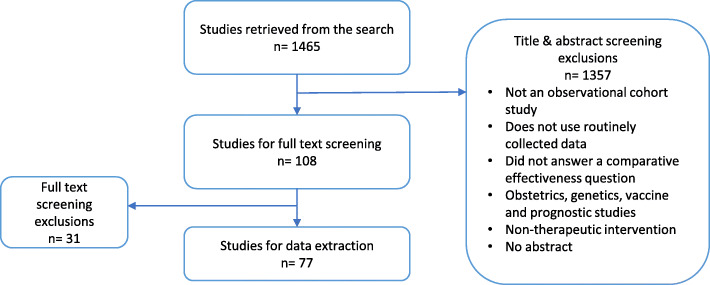
Study selection process

Among the 1465 articles retrieved from the search, 77 articles were selected for data extraction after screening for the title, abstract, and full text (Fig. 1).

Most of the studies were from North America and Europe and with a median sample size of 24,000 individuals. Ten articles (13%) did not report the study design. Fifty-three studies (69%) evaluated the pharmacological treatment. Forty-nine studies (63%) compared against active comparators. The sources of data were registry (*n* = 34/77, 44%), electronic health record (*n* = 17/77, 22%), administration data (*n* = 14/77, 18%), and health insurance claims (*n* = 20, 26%). Fifty-six percent of studies (43/77) received funding from not-for-profit organizations, and 13% (10/77) did not report the type of funding.

### Research transparency practices

Only seven articles (9%) mentioned the use of a reporting guideline. Fifty-three articles (69%) provided codes (e.g., ICD-10 codes) used to classify both exposures and outcomes. Ten articles (13%) indicated that data were available upon request (Table 2).

**Table 2:** Characteristics of included articles

	*N* = 77 (%)
**Name of journal**	
- *NEJM*	3 (4)
- *The Lancet*	2 (3)
- *JAMA*	13 (17)
- *Annals of Internal Medicine*	9 (11)
- *BMJ*	14 (18)
- *PLoS Medicine*	8 (11)
- *Circulation*	7 (9)
- *European Heart Journal*	4 (5)
- *Journal of the American College of Cardiology*	15 (19)
**Location of corresponding authors**	
- North America	40 (52)
- Europe	25 (32)
- Asia	10 (13)
- North American and Europe	1 (1)
- International	1 (1)
**Study design**	
- Cohort study	67 (87)
- Not clearly reported	10 (13)
**Treatment evaluated**	
- Pharmacological treatment	53 (69)
- Non-pharmacological treatment	23 (30)
- Both	1 (1)
**Comparator**	
- Active comparator	49 (63)
- Usual care	17 (22)
- No treatment	11 (14)
**Median sample size [min–max]**	24,000 [9100–80,000]
**Data source**^a^	
- Registry	34 (44)
- Electronic health record	17 (22)
- Health administration data	14 (18)
- Health insurance claims data	20 (26)
- Others	11 (14)
**Funding source**	
- Not for profit	43 (56)
- For profit	7 (9)
- Both	12 (16)
- No funding	5 (6)
- Unclear	10 (13)
**Research transparency practices**	
- Using a reporting guideline	7 (9)
- Code and algorithm used to classify exposures provided in supplementary documents	57 (74)
- Code and algorithm used to classify outcomes provided in supplementary documents	60 (78)
- A statement to provide data upon request	10 (13)

^a^One study might have more than one type of data sources

### Reporting essential information of the target trial 

Only 18% (*n* = 14/77) reported a diagram to illustrate the study design and reported the three essential time points (i.e., eligibility, treatment initiation, start of follow-up). Eighteen percent (*n* = 14/77) did not report completely essential time points, i.e., the start of follow-up, when individuals completed the eligibility criteria and when patients started the treatments of interest. Regarding the inclusion criteria, only 12% (*n* = 9/77) reported the exclusion of patients with contraindication to one of the evaluated interventions. Only one article explained the reason for not excluding patients with such a contraindication, due to the inability to identify these patients from the dataset. Sixty-five percent of articles (*n* = 50/77) did not specify the type of causal contrast estimated (Table 3).

**Table 3:** Reporting of essential information

Reporting of essential information	***N*** = 77 (%)
**Study characteristics**	
- Specification of the target trial	2 (3)
- Using a diagram to illustrate study design	14 (18)
**Eligibility criteria**	
- Inclusion criteria for the study	76 (99)
- Post-baseline events in inclusion criteria (e.g., use of treatment, no follow-up data)	9 (12)
- Exclusion of individuals with contraindications for interventions evaluated	9 (12)
**Adjustment for confounders**	
- Propensity score	60 (70)
- Inverse probability weighting	10 (12)
- Multivariable regression	15 (17)
- Instrumental variable	2 (2)
**Outcome**	
- Primary outcome reported	77 (100)
**Causal contrast of interests**	
- Intention-to-treat effect	11 (14)
- Per-protocol effect	6 (8)
- Both	10 (13)
- Not specified	50 (65)
**Key time point of the target trial**	
- Time point of the start of follow-up	72 (94)
- Time point of eligibility criteria	72 (94)
- Time point of treatment assignment	68 (88)
- All three time points	63 (81)

### Risk of bias due to failure of specifying a target trial

Overall, 33% (*n* = 25/77) raised concerns about the risk of bias. Of these, in one-fourth (*n* = 6/25), as the start of follow-up was not clearly reported, we could not determine if eligibility, treatment assignment, and the start of follow-up were synchronized (Fig. 1). In 76% (*n* = 19/25), the time when patients completed the eligibility criteria, initiated the treatments, and the start of follow-up was not aligned (Fig. 1). Among these 19 articles, in four articles (*n* = 4/19, 21%), the follow-up started when patients met eligibility but after patients initiated treatment (Table 1 (b)), which led to prevalent user bias and selection bias due to post-treatment eligibility [19–22]. The authors did not describe any solutions to address these biases in these four articles.

In seven articles (*n* = 7/19, 37%), the follow-up started when patients initiated treatment but before patients met the eligibility criteria leading to immortal time bias and selection bias due to post-treatment eligibility (Table 1 (c)) [23–29]. Among these, one article reported handling treatment exposure as a time-dependent variable to account for immortal time bias; however, this strategy was inadequate to account for selection bias due to post-treatment eligibility [25]. One article performed a sensitivity analysis to include participants who were excluded based on the post-treatment eligibility criteria and yielded similar results to the main analysis [27].

**Fig. 2:**
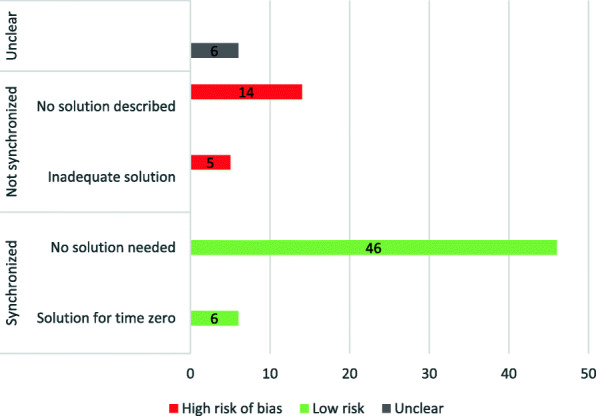
The number of studies at risk of bias due to lack of synchronization. Nineteen (25%) studies had a high risk of bias due to the lack of synchronization. Of these, 14 proposed no solution, and 5 used inadequate methods to address the bias. Six studies inadequately reported to enable the assessment of synchronization. Fifty-two (68%) studies had low risk of bias

In seven articles (*n* = 7/19, 37%), follow-up started when patients met the eligibility criteria, but patients were assigned to one of the treatment groups after the start of the follow-up, a situation both at risk of immortal time bias and misclassification of treatment (Table 1 (d)) [30–37]. Of these, four articles did not mention any solutions leading to high risk of selection bias [31, 32, 35, 37]; three articles treated treatment exposure as a time-dependent variable [30, 33, 36] which was inadequate to address the risk of misclassification, and one article randomly assigned individuals who had outcomes before treatment initiation to one of the two treatment groups [34] to mitigate the risk of bias. In one article (*n* = 1/19, 5%), individuals could start the treatment both before and after eligibility and the start of follow-up (Table 1 (b and d)); thus, the study was at risk of prevalent user bias and immortal time bias [38]. No solution was described in this article. Among these 19 articles that we identified biases, six articles (32%) discussed these biases in the limitations section (Fig. 2).

Table 4 presents the main features of 19 studies without synchronization of eligibility, treatment assignment, and follow-up.

**Table 4:** Studies without synchronization of eligibility, treatment assignment, and follow-up

Author, year	Patients (*n*)	Intervention	Comparator	Outcomes	Time points of eligibility	Times points of treatment assignment	Start of follow-up	Situations of failure of emulating a target trial	Type of bias might arise	Solution described by authors
Converse 2019 [19]	111	Angiotensin II inhibitors after continuous-flow left ventricular assist devices implant	Usual care	Gastrointestinal bleeding	30 days after operation to implant continuous-flow left ventricular assist devices	Within 30 days since operation	30 days after operation	b. Follow-up starts at eligibility but after treatment initiation.	Prevalent user bias, selection bias due to post-treatment eligibility	No solution described.
Skriver 2019 [20]	29,136	Low-dose aspirin	Usual care	Prostate cancer mortality	1 year after prostate cancer diagnosis	Within 1 year since diagnosis	1 year after prostate cancer diagnosis	b. Follow-up starts at eligibility but after treatment initiation.	Prevalent user bias, selection bias due to post-treatment eligibility	No solution described.
Friberg 2019 [21]	47,492	Oral anticoagulant	No treatment	Diagnosis of dementia, ischemic stroke, intracranial bleeding	At the time of diagnosis of atrial fibrillation	6 months before the start of follow-up	30 days after the diagnosis of atrial fibrillation	b. Follow-up starts at eligibility but after treatment initiation.	Prevalent user bias, selection bias due to post-treatment eligibility	No solution described.
Brauer 2019 [23]	424,996	Trazodone	Other antidepressants	Diagnosis of dementia	At the second prescription of antidepressant	At the first prescription of antidepressant	At the first prescription of antidepressant	c. Follow-up starts at treatment initiation but before eligibility.	Immortal time bias and selection bias due to post-treatment eligibility	No solution described.
Xie 2019 [24]	214,467	Proton pump inhibitors	H2 blockers	All-cause mortality	180 days after treatment group assignment	At the first prescription of either PPI or H2 blocker	At the first prescription of either PPI or H2 blocker	c. Follow-up starts at treatment initiation but before eligibility.	Selection bias due to post-treatment eligibility	No solution described.
Brown 2019 [25]	1555	Immunomodulatory disease-modifying therapies	No treatment	Disease progression	6 months after treatment commencement	Date of treatment commencement	Date of treatment commencement	c. Follow-up starts at treatment initiation but before eligibility.	Immortal time bias and selection bias due to post-treatment eligibility	Exposure was considered as time-dependent variable in all analysis to adjust for immortal time bias, but no solution described for selection bias due to post-treatment eligibility.
Kim 2019 [26]	1.0705	Statin	Statin + fenofibrate	Cardiovascular events	3 months after fenofibrate initiation	Date of fenofibrate initiation	Date of fenofibrate initiation	c. Follow-up starts at treatment initiation but before eligibility.	Immortal time bias and selection bias due to post-treatment eligibility	No solution described.
Axtell 2019 [30]	3276	Surgery	Medical therapy	All-cause mortality	The first echocardiographic diagnosis	Time of surgery	The first echocardiographic diagnosis	d. Follow-up start at eligibility, but treatment is assigned later.	Immortal time bias and misclassification of treatment	Time-dependent propensity score matching and allocate time before surgery to the control group.
Gharbi 2019 [31]	157,264	Antibiotic	No treatment	Bloodstream infection, hospital admission, and all-cause mortality	Date of urinary tract infection diagnosis	Within 7 days since diagnosis	Date of urinary tract infection diagnosis	d. Follow-up start at eligibility, but treatment is assigned later.	Immortal time bias and misclassification of treatment	No solution described.
Gray 2019 [32]	9653	Chemotherapy	Usual care	All-cause mortality and breast cancer mortality	Date of diagnosis	Date of chemotherapy commencement not reported but likely to be after the date of diagnosis	Date of diagnosis	d. Follow-up start at eligibility, but treatment is assigned later.	Immortal time bias and misclassification of treatment	No solution described.
van Rein 2019 [33]	272,315	Antithrombotic therapy	No treatment	Major bleeding	The date of their atrial fibrillation diagnosis	Treatment starts at any time after diagnosis	The date of their atrial fibrillation diagnosis	d. Follow-up start at eligibility, but treatment is assigned later.	Immortal time bias and misclassification of treatment	Exposure was treated as time-dependent variable in Cox regression.
Mahévas 2020 [34]	173	Hydroxychloroquine	Usual care	Survival without transfer to ICU	Admission to hospital	Within 48 h since admission	Admission to hospital	d. Follow-up start at eligibility, but treatment is assigned later.	Immortal time bias and misclassification of treatment	Patients from the control group who reached the primary outcome during the grace period were randomly assigned to one of the two groups, given that their observational data were compatible with both groups at the time of the event.
Rosenberg 2020 [35]	1438	Hydroxychloroquine with or without azithromycin	Usual care	In-hospital mortality	At admission to hospital	Start treatment at any time during hospitalization	24 h after admission	d. Follow-up start at eligibility, but treatment is assigned later.	Immortal time bias and misclassification of treatment	No solution described.
Geleris 2020 [38]	1446	Hydroxychloroquine	Usual care	Composite of intubation and death	24 h after arrival at the emergency department	Start treatment before or any time after hospitalization	24 h after arrival at the emergency department	b. Follow-up starts at eligibility but after treatment initiation for prevalent user group. d. Follow-up start at eligibility, but treatment is assigned later for new user group.	Prevalent user bias and immortal time bias	No solution described.
Jorge 2019 [36]	9659	Renal transplant	Usual care	All-cause mortality	The initial date of entry into the waitlist	Surgery at any time after being the waitlist	The initial date of entry onto the waitlist	d. Follow-up start at eligibility, but treatment is assigned later.	Immortal time bias and misclassification of treatment	Exposure was considered as time-dependent variable in all analysis.
Rea 2018 [28]	44,534	Two drug therapy antihypertension	Monotherapy antihypertension	Cardiovascular events	1 year after the dispensing of treatment	The first day of drug dispensing	The first day of drug dispensing	c. Follow-up starts at treatment initiation but before eligibility.	Immortal time bias and selection bias due to post-treatment eligibility	No solution described.
Lin 2018 [27]	6558	Low-dose of rivaroxaban	Standard dose of rivaroxaban	Major bleeding events	Patients refilled prescription more than once since the start of rivaroxaban	The first prescription of rivaroxaban	The first prescription of rivaroxaban	c. Follow-up starts at treatment initiation but before eligibility.	Immortal time bias and selection bias due to post-treatment eligibility	A sensitivity analysis was performed to include patients who did not refill their prescription after the first one.
Siontis 2018 [22]	25,523	Apixaban or switching from warfarin to apixaban	Warfarin	Ischemic stroke, major bleeding events, and death	Diagnosis of atrial fibrillation	The date of the initial anticoagulation prescription or the date of apixaban prescription if patient switched from warfarin to apixaban	The date of the initial anticoagulation prescription or the date of apixaban prescription if patient switched from warfarin to apixaban	b. Follow-up starts at eligibility but after treatment initiation.	Selection bias due to post-treatment eligibility	No solution described.
Ramos 2018 [29]	46864	Statin	No treatment	Incidences of atherosclerotic cardiovascular diseases and all-cause mortality	The second invoice of statin	The first invoice of statin	The first invoice of statin	c. Follow-up starts at treatment initiation but before eligibility.	Immortal time bias and selection bias due to post-treatment eligibility	No solution described.

## Discussion

Our review showed that 20% (*n* = 14/77) of the articles did not adequately report essential information of the study design. A third of reviewed articles had unclear risk of bias or high risk of selection bias and/or immortal time bias due to the choice of the time of eligibility, treatment assignment, and the start of follow-up that failed to mimic the randomization. In only 25% of the articles at risk of bias, a solution was described; however, these solutions were not adequate to eliminate the risk of bias completely. The lack of synchronization arises when investigators want to select individuals who might have better treatment adherence, i.e., select only individuals who adhered to the treatment for a given period (Table 5 (c)), or only individuals who have adhered to the treatment for a given period are classified as exposed (Table 5 (d)). To address the selection bias caused by using a post-treatment event to include individuals or predict treatment strategies in the future, Hernan et al. proposed creating a clone, i.e., an exact copy of the population, assign them to one of the treatment groups and censor when they deviate from the assigned treatment [12].

**Table 5:** Solutions proposed by Hernan et al. to address the risk of bias when time points of eligibility, treatment assignment, and the start of follow-up are not aligned

Situations	Possible solutions
*a. Follow-up starts after eligibility criteria completion and treatment assignment which leads to prevalent user bias.*	Select new users [12].
*b. Follow-up starts at eligibility but after treatment assignment which leads to prevalent user bias and selection bias due to post-treatment eligibility.*	Select new users and ensure that individuals are not selected by an event that happens after the follow-up starts [12].
*c. Follow-up starts before treatment assignment and eligibility which leads to immortal time bias and selection bias due to post-treatment eligibility.*	Keep all individuals who start the treatment since the start of follow-up, create an exact copy of the population, assign them to one of the intervention groups from the start of the follow-up, and censor when they start to deviate from assigned treatment [39]. One strategy which is often used to account for immortal time bias in literature is to consider exposure as a time-dependent variable. However, this strategy is not adequate to address the risk of selection bias due to post-treatment eligibility, as an uncensored group might not be exchangeable with the censored group [3].
*d. Follow-up starts at eligibility, but treatment is assigned later which leads to immortal time bias and misclassification of treatment.*	1) Randomly assign individuals to one of the treatment strategies [12]. 2) Create an exact copy of the population, assign them to one of the intervention groups from the start of the follow-up, and censor when they start to deviate from assigned treatment [39]. One strategy which is often used to account for immortal time bias is to consider exposure as a time-dependent variable. However, this strategy is inadequate to address the risk of misclassification, because if individuals have outcomes during the grace period, we are uncertain which intervention group they should be classified into.

Another common reason for the lack of synchronization in observational studies using routinely collected data is due to having a grace period, i.e., individuals start to use treatment within a given period after the start of follow-up and eligibility (Table 5 (d)); thus, investigators can increase the number of eligible individuals. For example, to compare the effectiveness of hydroxychloroquine versus standard of care in the treatment for COVID-19 patients, the number of patients who initiated hydroxychloroquine immediately after hospital admission would be quite low. To increase the number of eligible patients for the analysis, investigators allowed for a grace period to assign patients who started hydroxychloroquine within 48 h since admission to the intervention group [34, 35]. However, a challenge of having a grace period is that we could not assign patients to one of the intervention groups at the start of the follow-up as in an RCT. If a patient had an outcome within 48 h since admission, it is uncertain if they should be classified as exposed or control group. To overcome the challenge of having a grace period, Hernan et al. also recommended following the strategy as above, i.e., to create an exact copy of the population, assign them to one of the intervention group, censor when they start to deviate from assigned treatment, and use inverse probability weighting to adjust for post-treatment censoring bias [12, 39] (Table 5). However, the use of such an approach was never reported in our sample. Although Hernan et al. proposed this approach in 2016, there are only a few studies applying this approach due to methodological and logistical challenges. Maringe et al. provided a detailed tutorial to perform the cloning strategy [40].

Additionally, the emulated trial framework highlights the importance of the new-user design by identifying all eligible in the defined population who started the study treatments to avoid these biases. The selection of only new users, however, might reduce the sample size and the study power [41, 42]. To address this challenge, sensitivity analysis could be used to assess the magnitude of potential bias related to including prevalent users [41, 42].

Furthermore, some other essential information was missing in the report of observational studies in our sample, particularly specifying if patients with contraindication with one of the evaluated treatments were excluded from the analysis. This issue could be problematic as we are uncertain if patients in different treatment groups were comparable. For example, in a study, patients who had contraindication with evaluated treatments were classified as the control group [43]. It means that patients in the intervention and control groups were not exchangeable, which violated a fundamental condition of causal inference.

Previous studies have also highlighted the incomplete reporting and potential bias in the implementation of observational studies. Luijken et al. found that 6% of the evaluated observational studies did not specify if new users or prevalent users were included, and in only half of the studies using new user design, time point of eligibility, treatment initiation, and start of follow-up were synchronized [44]. Due to these avoidable methodological pitfalls, the results of observational studies could be biased and mislead healthcare decisions [45]. The emulated trial framework which relies on synchronization of eligibility, treatment assignment, and the start of follow-up to mimic the randomization of RCT can help in reducing the risk of bias. However, the approach proposed by Hernan has also some limitations particularly in some situations, synchronization of the time points of the eligibility criteria, start of treatment, and start of follow-up is not feasible. By explicitly reporting these components and the decision made when emulating the target trials, researchers could help readers in assessing the extent that results might be influenced by bias and whether the choice of methodology to address this bias was appropriate to ensure the validity of results. We propose a checklist following the framework of emulated trials to help readers and reviewers to identify the common pitfalls of observational studies (Table 6).

**Table 6:** Checklist to determine the potential risk of bias in observational studies

Guiding question	Explanation
1. When does the follow-up start?	- Check if the authors report the start of follow-up. It might be called the baseline, index date, and time zero.
2. When do individuals complete eligibility?	- Check if authors report when individuals should complete eligibility.
2.a. Can individuals be eligible at multiple times?	- Check if individuals could be eligible at multiple times and whether authors used a strategy to overcome this: (1) choose a single eligible time and (2) choose all eligible times and conduct a sequence of trials at each eligible time.
2.b. Is there any post-baseline event (i.e., an event after the follow-up starts) in the eligibility criteria?	- Check if any events after the start of follow-up are listed in the eligibility criteria, e.g., complete 2 consecutive prescriptions or no outcome for the first 2 months after the start of follow-up.
3. When are individuals assigned to an exposed or non-exposed group?	- Check if the authors report clearly when individuals are classified as exposed or non-exposed group.
3a. Do individuals have to use treatment for a given period to be classified as an exposed group?	- Check if individuals have to use treatment for a given period, e.g., complete 2 consecutive prescriptions to be classified as exposed and non-exposed, if not, start the treatment or complete only 1 prescription.
3.b. Is there a grace period?	- Check if individuals can start the treatment sometime after the start of follow-up and eligibility.

Our study has some limitations. First, to ensure the feasibility of the study, we restricted the search to high impact factor journals, which might underestimate the prevalence of bias due to the lack of synchronization of eligibility, treatment assignment, and start of follow-up. However, our aim is to raise awareness of the common problems of reporting and conducting observational studies using RCD that need to be addressed in future research. Second, we were unable to determine the magnitude of the bias. For example, if there are more individuals who have outcomes during the grace period, the effect estimates would be at higher risk of bias, because these individuals are more likely to be classified in the control group. Third, we did not evaluate the risk of confounding in the included studies. Nevertheless, the emulated trial framework and the cloning strategy can address the confounding bias.

## Conclusions

In conclusion, reporting of essential information of the study design in observational studies remained suboptimal. The lack of synchronization of eligibility, treatment assignment, and the start of follow-up is common among observational studies, which leads to different types of bias such as prevalent user bias, immortal time bias, and selection bias due to post-treatment eligibility. Researchers and physicians should critically appraise the results of observational studies using routinely collected data.

## Supplementary Information

**Additional file 1: Table S1.** Search strategy. **Table S2.** Eligibility criteria. **Table S3.** Data extraction form. **Table S4**. Data Extraction Form Explanation.

## Data Availability

The dataset is available at 10.5281/zenodo.5543469.

## Abbreviations

PRISMA: Preferred reporting items for systematic reviews and meta-analyses
RCD: Routinely collected data
RCTs: Randomized controlled trials
RECORD-PE: Reporting Guideline for observational studies using routinely collected data for pharmacoepidemiology
