# Supplementary material for: Risk of bias in observational studies using routinely collected data of comparative effectiveness research: a meta-research study
Source: BMC Med. 2021 Nov 23;19:279. doi: 10.1186/s12916-021-02151-w (PMC8608432; doi:10.1186/s12916-021-02151-w)
Supplement: Supplementary file 1 — Additional file 1: Table S1. Search strategy. Table S2. Eligibility criteria. Table S3. Data extraction form. Table S4. Data Extraction Form Explanation. [file 12916_2021_2151_MOESM1_ESM.docx]

**Table S1. Search strategy**

| 1. HIF journals:   (((Vital statistics[MESH] OR Survival Analysis[MESH] OR mortality[tiab] OR survival[tiab] OR initiator*[tiab] OR non-initiator*[tiab] OR user*[tiab] OR free survival[tiab] OR person-year[tiab] OR g-methods OR g estimation OR instrumental variable OR propensity score[MESH] OR propensity score* OR compar*[tiab] OR effectiveness[tiab] OR causal inference*[tiab] OR causal effect*[tiab] OR effectiveness[tiab] OR evaluat*[tiab])) AND ((((((((Observational Study[Publication Type]) OR Observational Stud*[MeSH Terms]) OR Observational Stud*[Title/Abstract]) OR cohort studies[MeSH Terms]) OR retrospective[Title/Abstract]) OR longitudinal[Title/Abstract])))) AND (((((((("N Engl J Med"[Journal]) OR "Lancet"[Journal]) OR "JAMA"[Journal]) OR "BMJ"[Journal]) OR "Ann Intern Med"[Journal]) OR "BMC Medicine"[Journal]) OR "PLOS Medicine"[Journal])) filter: 2018/06/01 to 2020/06/30 |
| --- |
| 1. Specialty Journals:   (((Vital statistics[MESH] OR Survival Analysis[MESH] OR mortality[tiab] OR survival[tiab] OR initiator*[tiab] OR non-initiator*[tiab] OR user*[tiab] OR free survival[tiab] OR person-year[tiab] OR g-methods OR g estimation OR instrumental variable OR propensity score[MESH] OR propensity score* OR Comparative effectiveness research[MESH] OR compar*[tiab] OR effectiveness[tiab] OR causal inference*[tiab] OR causal effect*[tiab] OR effectiveness[tiab] OR evaluat*[tiab])) AND (Observational Study[Publication Type] OR Observational Stud*[MeSH Terms] OR Observational Stud*[Title/Abstract] OR cohort studies[MeSH Terms] OR retrospective[Title/Abstract] OR longitudinal[Title/Abstract])) AND ("Lancet Diabetes & Endocrinol."[Journal] OR "Diabetes Care"[Journal] OR "Diabetes "[Journal] OR "European Heart Journal"[Journal] OR "J Am Coll Cardiol"[Journal] OR "Circulation"[Journal]) filter: 2018/06/01 to 2020/06/30 |

**Table S2. Eligibility criteria**

| **Include** | **Exclude** |
| --- | --- |
| Comparative | RCTs |
| Therapeutic interventions | Case-control, systematic reviews, descriptive studies, etc. |
| Routinely collected data | Non-therapeutic interventions: large scale public health interventions, diet, following guidelines, policy recommendations, etc. |
|  | Genetics, obstetrics, prognostic, diagnostic, vaccine studies, etc. |
|  | Secondary analysis of data collected from research purposes |
|  | No abstract |

**Table S3. Data extraction form**

**Data Extraction Form:**

***Description/characteristics of the study***

Reviewer name: ­­________________________

Search group:

- General Medicine Journals
- Specialty Journals

Authors: ________________________

Study name: ­­________________________

Year of Publication: ________________________

Journal name: ________________________

DOI: ________________________

1. Location of corresponding author:

| - Europe - North America - Asia | - South America - Oceania - Unclear | - Other: ____________________________ |
| --- | --- | --- |

1. Funding source:

| - Private - Public - None | - Both - Unclear |
| --- | --- |

1. Medical area:

| - Cardiology - Rheumatology - Endocrinology - Oncology | - Infectious Disease - Psychiatry/Psychology - Neurology - Respiratology | - Unclear - Other: ________________________ |
| --- | --- | --- |

1. Type of intervention:

- Pharmacological
- Non-pharmacological
- Both

1. Data source:

| - Registry - Health administration data - Surveillance data - Electronic health record | - Pharmacy data - Insurance data - Other: ________________________ |
| --- | --- |

***Reporting***

1. Did they report following any reporting guidelines?

- Yes
- No
  1. If YES, which guideline? ________________________

1. Was the objective of the study reported?

- Yes
- No
  1. If YES, what was the objective? ________________________

1. *Study Design*:
   1. Was the study design reported?

- Yes
- No
  1. If YES, what is it? ________________________
  2. Do they use a diagram to illustrate key aspects of the study design?
- Yes
- No

1. *Setting*:
   1. Was the setting of the study reported? (ie. hospital based, community based, etc.)

- Yes
- No
  1. Was the period of data collection recorded?
- Yes
- No
  1. Was the location reported?
- Yes
- No
  1. Was the follow-up period reported?
- Yes
- No

1. *Eligibility*:
   1. Was the eligibility criteria reported?

- Yes
- No
  1. If YES, what was the eligibility criteria? ________________________
  2. Did they specify users with specific indications?
- Yes
- No
  1. Did they specify exclusion of patients due to contraindications?
- Yes
- No
  1. Did they specify if patients were included once or multiple times?
- Yes
- No
  1. Did they report the code or algorithm used to select patients?
- Yes
- No

1. *Treatment Strategies*:
   1. Did they describe the treatment strategy/strategies?

- Yes
- No
  1. If YES, what were the treatment strategies? ________________________
  2. Did they specify the data source from which the treatment information was obtained?
- Yes
- No
  1. If YES, what was/were the data source(s)? ________________________
  2. Did they report how and when patients initiate treatment?
- Yes
- No
  1. Did they report a time window to initiate the intervention? (grace period)
- Yes
- No
- Not applicable
  1. Did they specify the comparator(s)?
- Yes
- No
  1. If YES, what kind of comparator(s) was it?
- No treatment
- Usual care
- Active comparator(s)
  1. If ACTIVE COMPARATOR(S), was there a time window for initiation to consider?
- Yes
- No
  1. Did they outline an approach to handle more than one relevant drug exposure?
- Yes
- No
- Not applicable

1. *Follow-up:*
   1. Did they specify the start of the follow-up?

- Yes
- No/Unclear

1. *Outcome:*
   1. Did they report the outcome of interest?

- Yes
- No
  1. Did they report the code and algorithm to classify the outcome?
- Yes
- No

***Conduct***

1. Did the author(s) specify a target trial?

- Yes
- No

1. *Treatment assignment:*
   1. Was there a grace period?

- Yes
- No (if no, you can skip to question 15.5)
  1. If YES, what was the grace period? ________________________
  2. If there was a grace period, was there a risk of misclassification of treatment?
- Yes
- No
  1. If there was a risk of misclassifying the treatment, how was it addressed?
- Creating clone for each strategy and censor when patients deviate from the assigned treatment
- No solution described
  1. Does treatment assignment depend on any prognostic factor? (time varying treatment)
- Yes
- No
  1. If YES, how was it addressed?
- Randomly assign the individual to one of the strategies
- Clone exact copies of individuals and assign each clone to one of strategies then censor clone when strategies are deviated from. Adjustments needed for post-time zero selection bias (e.g., via IP weighting)
- No solution described
- Other: _________________________________

1. *Defining time zero:*
   1. When does follow-up start for a patient (baseline)? ________________________
   2. When do patients initiate the treatment (treatment assignment)? ________________________
   3. When do patients meet the complete eligibility criteria? ________________________
   4. Was time zero, treatment assignment and completed eligibility synchronized?

- Yes
- No
- Unclear
  1. If NO, choose one of the scenarios below: *T0= time zero (dotted line), E= eligibility criteria, A= treatment strategy
- **A)** Time zero is set after eligibility and strategy assignment


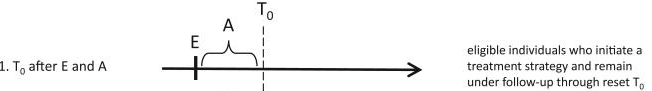


- **B)** Time zero is set at eligibility but after strategy assignment


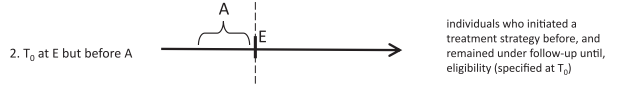


- **C)** Time zero is set before eligibility


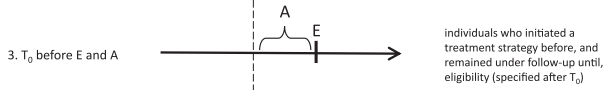


- **D)** Time zero is set at eligibility, but treatment is assigned after time zero


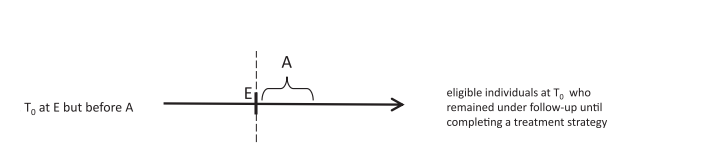


- 1. If time zero, treatment assignment and completed eligibility were NOT synchronized, what bias was introduced?
- Selection bias
- Immortal time bias
  1. Did the authors have a strategy to address the bias introduced?
- Yes
- No
- Unclear
  1. If YES, what was the strategy? ________________________
  2. Was time zero hard to define? (i.e. Patients can have time zero at multiple time points.)
- Yes
- No
- Unable to determine/Unclear
  1. If YES, how was it addressed?
- Choose one of the multiple times as time zero (the first time zero or a randomly chosen one)
- Choose all eligibility times as time zero and conduct a sequence of nested trials
- Choose some of the eligible times as time zero and match person-time when initiation occurs with person-time when no initiation occurs.
- No solution described
  1. Was there a problem of treatment adherence?
- Yes
- No
- Unclear
  1. If YES, how was it addressed?
- Estimate the per-protocol effect by defining time zero at treatment assignment and censoring when patients deviate from assigned strategy. Adjustments needed for confounding at time zero and post-time zero censoring.
- No solution described
  1. For the statistical analysis, did they use:
- Per-protocol analysis
- Intention-to-treat analysis
- Both
- Other: ___________________
  1. Were adjustments made for baseline confounding factors?
- Yes
- No
  1. If YES, which one(s):

| - Matching with or without propensity score - Stratification or regression with or without propensity score - Inverse probability weighting | - Standardization - G-estimation - Other: _______________ |
| --- | --- |

***Discussion/Conclusion:***

1. *Discussion/Conclusion*:
   1. Did the author discuss the limitations of the study?

- Yes
- No
  1. Does the conclusion include causal language (causal vs association)?
- Yes
- No
  1. Does the conclusion or discussion include any spin?
- Yes
- No
  1. Have authors suggested conducting future studies?
- Yes
- No
  1. If YES, what kind of study/studies?
- Randomized control trial
- Replication studies
- Other observational studies
- Further research
- Other: _____________________

*Extra Notes/Comments:*

**Table S4. Data Extraction Form Explanation**

| **Section** | **No.** | **Question** |  | **Explanation** |
| --- | --- | --- | --- | --- |
| ***Description/Characteristics of the study*** | | | | |
|  | I | Reviewer name |  | The name of the reviewer |
|  | II | Search group |  | The high impact factor journal categories |
|  | III | Authors |  | The main author(s) of the article |
|  | IV | Study name |  | The title of the study |
|  | V | Year of publication |  | The year of publication |
|  | VI | Journal name |  | The name of the journal |
|  | VII | DOI |  | The DOI (Digital Object Identifier) of the article |
|  | 1 | Location of corresponding author |  | The location of the authors of the study |
|  | 2 | Funding source |  | The type of funding source |
|  | 3 | Medical area |  | The general medical area of interest in the study. Specifically the field related to the treatment. |
|  | 4 | Type of intervention |  | The type of intervention studied |
|  | 5 | Data source |  | The type of data source |
| ***Reporting*** | | | | |
| Reporting guidelines | 6  6.1 | Did they report following any reporting guidelines?  If YES, which guideline? |  | State whether the article reported the use of any reporting guidelines and specify the guideline(s) mentioned. |
| Objectives | 7  7.1 | Was the objective reported?  If YES, what was the objective? |  | State whether the article reported specific objectives, including any pre-specified hypotheses. If so, describe the objective. |
| Study Design (8) | 8.1  8.2 | Was the study design reported?  If YES, what is it? |  | State whether the article reported the study’s design with a commonly used term. If so, state the study design indicated. |
|  | 8.3 | Do they use a diagram to illustrate key aspects of the study design? |  | State whether the article used a diagram(s) to illustrate key aspects of the study design(s), including exposure, washout, lag and observation periods, and covariate definitions as relevant? Does not include diagrams on how the study population was selected. |
| Setting (9) | 9.1 | Was the setting reported? (i.e. hospital based, community based, etc.) |  | State whether the general setting of the study was described. For instance, a study done in hospital setting or a community based setting. |
|  | 9.2 | Was the period of data collection recorded? |  | State whether the article reported the period of time in which the data were collected for the study. |
|  | 9.3 | Was the location reported? |  | State whether the article reported the specific location(s) of the study. |
|  | 9.4 | Was the follow-up period reported? |  | State whether the article indicated the follow-up period for the study. |
| Eligibility (10) | 10.1  10.2 | Was eligibility criteria reported?  If YES, what was the eligibility criteria? |  | State whether the article described the criteria for entry into the study to identify the study population. If so, describe the eligibility criteria. |
|  | 10.3 | Were post baseline events included in the inclusion criteria? |  | If there were any post baseline events in the inclusion criteria e.g. using treatment for certain time, no outcome for certain time |
|  | 10.4 | Was the time point of eligibility reported? |  | When individuals fulfilled all criteria to be included in the analysis |
|  | 10.5  10.6 | Was time zero hard to define?  If YES, how was it addressed? |  | State whether time zero or start of follow-up was difficult to define. Time zero is difficult to define when an individual could meet eligibility at multiple times for a patient leading to a patient with multiple time zeros. |
|  | 10.7 | Did they specify users with specific indications? |  | Specify whether only users with a specific indication were included. (i.e. A study where patients were selected with a specific medical condition being treated as opposed to selecting all patients regardless of the medical condition.) |
|  | 10.8  10.9 | Did they specify exclusion of patients due to contraindications?  Was a justification for including patients with contraindication reported? |  | State whether the study excluded patients due to contraindications to the treatment. |
|  | 10.10  10.11 | Were there differences in the time period for eligibility?  If YES, what was the solution? |  |  |
|  | 10.12 | Did they specify if patients were included once or multiple times? |  | Specify whether patients were allowed to enter the study population once or if multiple entries were permitted. (i.e. new user study design, etc.) |
|  | 10.13 | Did they report the code or algorithm used to select patients? |  | State whether the study details the diagnostic codes or algorithms used to identify subjects. |
| Treatment Strategies (11) | 11.1  11.2 | Did they describe the treatment strategy?  If YES, what were the treatment strategies? |  | State whether the study clearly defined all treatment strategies including a description of how the drug exposure definition was developed. If so, describe the treatment strategies. |
|  | 11.3  11.4 | Did they specify the data source from which the treatment information was obtained?  If YES, what was/were the data source(s)? |  | State whether the article specified the data source(s) used to obtain treatment information including drug dispensation. If so, specify the data source(s) used. |
|  | 11.5 | Did they report how and when patients initiate treatment? |  | State whether the article specified how and when patients initiate the treatment strategy. |
|  | 11.6 | Did they report a time window to initiate the intervention? (grace period) |  | State whether the study described a time window(s) during which an individual is considered exposed to the drug(s) i.e. a grace period. Grace period is defined as a time period from the completion of eligibility to the start of treatment where the patient is considered exposed. |
|  | 11.7  11.8  11.9 | Did they specify the comparator(s)?  If YES, what kind of comparator was it?  If active comparator, was there a time window for initiation to consider? |  | State whether the article clearly defined all comparators. No treatment refers to the patient received no treatment at all. Usual care refers to the patient receiving some usual treatment for their medical condition but not the treatment(s) of interest in the study. Active comparator refers to a known, effective treatment (as opposed to a placebo) to be compared to an experimental treatment. |
|  | 11.10 | Did they outline an approach to handle more than one relevant drug exposure? |  | State whether the article detailed an approach to handle more than one relevant drug exposure, including but not limited to a wash out period. |
| Follow-up (12) | 12.1 | Did they specify the start of the follow-up? |  | State whether the article reported the start of the follow-up period. This includes if it is clear in the article but not explicitly stated. |
| Outcomes (13) | 13.1 | Did they report the outcome of interest? |  | State whether the study clearly defined the outcomes of interest including a description of how the outcome definition was developed, if relevant. |
|  | 13.2 | Did they report the code and algorithm to classify the outcome? |  | State whether the study details the diagnostic codes or algorithms used to identify subject outcomes. |
| ***Conduct*** | | | | |
| Target trial | 14 | Did the author(s) specify a target trial? |  | State whether the study explicitly specified a target trial for emulation. |
| Treatment assignment (15) | 15.1  15.2 | Was there a grace period?  If YES, what was the grace period? |  | State whether the study had a grace period. If so, specify the grace period.  Grace period is defined as a time period from the completion of eligibility to the start of treatment where the patient is considered exposed. During the grace period a patient can be in either treatment group thus if the outcome occurs, it is unclear what group should they be counted in. There is a risk of misalignment and introduction of bias. |
|  | 15.3  15.4 | If there was a grace period, is there risk of misclassification of treatment?  If there was a risk of misclassifying the treatment, how was it addressed? |  | If there is a grace period, state whether there is a risk of misclassifying the patient into the wrong treatment group. If there was a risk of misclassification, state if and how it was addressed.  Example: *A study on antibiotics*  A risk of misclassification can occur even when there is a treatment group for the grace period patients. Take a study with these three treatment groups: antibiotic, deferred antibiotic, no treatment. There is a risk of misclassification between the deferred antibiotic and no treatment groups as a patient, during the 7 day grace period, could be classified as either group until the patient starts the antibiotic. |
| Defining time zero (16) | 16.1 | When does follow-up start (baseline)? |  | Specify the start of follow-up (i.e. time zero or T0). |
|  | 16.2 | When were patients assigned to the treatment? |  | Specify when patients were assigned to the treatment strategies (i.e. treatment assignment or A) |
|  | 16.3 | When do patients meet the complete eligibility criteria? |  | Specify when patients meet the complete eligibility criteria (i.e. completion of eligibility criteria or E) |
|  | 16.4 | Were time zero, treatment assignment and completed eligibility synchronized? |  | State whether the start of follow-up, the initiation of the treatment strategy and completion of the eligibility criteria are synchronized.  Note: If the start of follow-up is set for 1 day after cohort entry (completion of eligibility criteria) the three are not synchronized but bias is not introduced. |
|  | 16.5 | If NO, choose one of the scenarios below:  A) Time zero is set after eligibility and strategy assignment  B) Time zero is set at eligibility but after strategy assignment  C) Time zero is set before eligibility  D) Time zero is set at eligibility, but treatment is assigned after time zero |  | If the time zero, treatment assignment and completion of eligibility are NOT synchronized, state which scenario the study falls into:  A - Start of follow-up set after eligibility completion and treatment assignment aka the eligible patient starting treatment before the start of follow-up and continuing it after. This may lead to a left truncated follow-up which leads to selection bias.  B – Patient initiates treatment strategy before the start of follow-up and all patients included in the analysis are required to meet some eligibility criteria at the start of follow-up. This analysis would only include patients who remained under follow-up who also meet these post-treatment criteria. This leads to selection bias.    C - This occurs when several sequential criteria must be met to complete eligibility criteria. Patients are assigned a treatment strategy based on the observed data before eligibility is fully met and follow-up begins at treatment assignment. When treatment assignment predates eligibility, selection bias may arise. By definition, the outcome cannot occur between treatment assignment and completion of eligibility. This leads to immortal time bias.  D - Individuals are assigned to a treatment strategy based on what treatment they happened to use after time zero (post-baseline criteria) as opposed to assigning individuals to a treatment strategy based on their data at time zero. This leads to immortal time bias. |
|  | 16.6  16.7  16.8 | If time zero, treatment assignment and completed eligibility were NOT synchronized, what bias could be introduced?  Did the authors have a strategy to deal with the bias introduced?  If YES, what was the strategy? |  | If the three are not synchronized, state what bias was introduced based on the explanation of question 16.5. Depending on the bias, state if there was a solution used to manage this bias and what the solution(s) were. |
|  | 16.9  16.10 | Was there a problem of treatment adherence?  If YES, how was it addressed? |  | State whether the study had issues with patients adhering to treatment (i.e. crossover, etc.) |
|  | 16.11 | For the statistical analysis, did they use: |  | State what statistical analysis was used in the study (i.e. per-protocol, intention-to-treat, both, not specified, or other). |
|  | 16.12  16.13 | Were adjustments made for baseline confounders?  If YES, which one(s): |  | State which technique was used specifically to account for baseline confounding. Adjusting on baseline confounders ensures comparability (exchangeability) of the groups and is a form of emulation of randomization. |
|  | 16.14  16.15 | Does treatment assignment depend on any prognostic factor? (time-varying treatment)  If YES, how was it addressed? |  | State whether prognostic factors that change over time affect whether or how treatment is assigned (i.e. the decision of whether patients receive treatment or not is based on severity, a biomarker, or a prognostic test result that is itself affected by past treatment). This can lead to time-varying confounding. If there was a risk of time-varying confounding, state how was it addressed.  Example:  Examining the effect of testosterone treatment on risk of acute myocardial infarction. Time varying confounding may occur by the change in serum testosterone levels over time, which in turn could affect treatment patterns in the future. |
| ***Discussion/Conclusion*** | | | | |
| Discussion/ Conclusion (17) | 17.1 | Did the author discuss the limitations of the study? |  | State whether the article reports the limitations of the study. |
|  | 17.2 | Does the conclusion include causal language? (causal vs association) |  | State whether the conclusion section used causal language or language of association to summarize the results. |
|  | 17.3 | Does the conclusion or discussion include any spin? |  | State whether the conclusion includes any spin (i.e. authors concluding without acknowledgment of the results for instance the outcome is negative, but they conclude as if it's positive, or authors conclude by using secondary outcomes and not the primary outcomes) |
|  | 17.4  17.5 | Have authors suggested conducting future studies?  If YES, what kind of study/studies? |  | State whether the article suggests the conduct of future research. If so, state what kind of studies were suggested. |
|  |  | Extra Notes/Comments |  | Detail any extra notes or comments that were not addressed in the data extraction form. |
|  |  | Data availability |  | A statement that data is available and accessible |
|  |  | Sample size |  | Number of patients included in the analysis |
